# Revealing Gender Bias from Prompt to Image in Stable Diffusion †

**DOI:** 10.3390/jimaging11020035

**Published:** 2025-01-24

**Authors:** Yankun Wu, Yuta Nakashima, Noa Garcia

**Affiliations:** D3 Center, Osaka University, Suita 565-0871, Osaka, Japan; n-yuta@ids.osaka-u.ac.jp (Y.N.); noagarcia@ids.osaka-u.ac.jp (N.G.)

**Keywords:** gender bias, bias evaluation, text-to-image generation

## Abstract

Social biases in generative models have gained increasing attention. This paper proposes an automatic evaluation protocol for text-to-image generation, examining how gender bias originates and perpetuates in the generation process of Stable Diffusion. Using triplet prompts that vary by gender indicators, we trace presentations at several stages of the generation process and explore dependencies between prompts and images. Our findings reveal the bias persists throughout all internal stages of the generating process and manifests in the entire images. For instance, differences in object presence, such as different instruments and outfit preferences, are observed across genders and extend to overall image layouts. Moreover, our experiments demonstrate that neutral prompts tend to produce images more closely aligned with those from masculine prompts than with their female counterparts. We also investigate prompt-image dependencies to further understand how bias is embedded in the generated content. Finally, we offer recommendations for developers and users to mitigate this effect in text-to-image generation.

## 1. Introduction

Text-to-image generation has shown a superior capability for generating high-fidelity images. Given natural language inputs, known as prompts, cutting-edge models such as Stable Diffusion [1] and DALL-E 2 [2] produce high-quality images that align closely with the given prompts. However, their widespread accessibility and diverse applications across various domains have raised ethical concerns, such as the social impact of data [3,4,5], bias [6,7,8], privacy [9,10], or intellectual property issues [11,12]. Evaluating these problems remains a relatively underexplored challenge. In this work, we focus on developing an evaluation protocol for gender bias in Stable Diffusion models.

**Figure 1:**
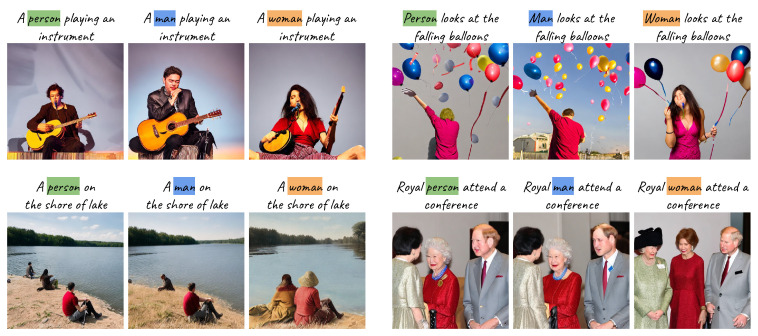
We use free-form triplet prompts to analyze the influence of gender indicators on the overall image generation process. We show that (1) gender indicators influence the generation of objects (**left**) and their layouts (**right**), and (2) the use of gender *neutral* words tends to produce images more similar to those prompted by *masculine* indicators rather than *feminine* ones.

It has been widely shown that certain adjectives [7] or professions [7] can lead to the generation of stereotypical demographic attributes in faces. However, disparities according to gender are also shown in regions beyond the faces, which are intended to fill the images [6]. Figure 1 shows triplets of generated images from prompts that differ only in the gender indicators (gender indicators refer to words that indicate the gender of a person). We observe that while the representation of the faces changes accordingly, unexpected variations also occur in other parts of the images, even when not explicitly mentioned in the prompt. For example, differences can be seen in the object depicted (e.g., different musical instruments on the upper-left image) and the layout of the image (e.g., on the right images). This suggests that gender bias extends beyond face representations and influences the broader context of the entire image. Most previous works [4,6,7,8,13,14,15,16] report demographic bias focused on generated faces in text-to-image generation [6,7,13,17], often neglecting to examine the generation process and how bias perpetuates from prompt to image.

In this paper, we investigate the internal components of Stable Diffusion to uncover the origins of gender bias and how it pertains. We suggest that these disparities arise from the interplay of representational disparities and prompt-image dependencies during image generation: the process involves transitioning from prompt space to image space, potentially treating genders differently and resulting in representational disparities. To analyze differences regarding genders, we set triplet prompts that differ only in gender indicator, and quantify representational disparities (Section 4) and prompt-image dependencies (Section 6). Our automatic evaluation protocol allows us to formulate and answer the following research questions (RQ):

**RQ1**  Do images generated from neutral prompts exhibit greater similarity to those generated from masculine prompts than to images generated from feminine prompts and, if so, why?
**RQ2**  Do object occurrences in images significantly vary based on the gender specified in the prompt? If there are differences, do these object occurrences from neutral prompts exhibit greater similarity to those from masculine or feminine prompts?
**RQ3**  Does the gender in the input prompt influence the prompt-image dependencies in Stable Diffusion, and if so, which prompt-image dependencies are more predisposed to be affected?

We conduct experiments on three versions of Stable Diffusion models. The template-free natural language prompts are derived from four caption datasets and a text set generated by ChatGPT [18]. Despite differing only in the gender indicator, the triplets exhibit a consistent trend across all Stable Diffusion models. Our key findings include the following:

- The images generated from neutral prompts are consistently more similar to those from masculine prompts than feminine prompts.
- Across *all internal stages of the generation process*, representation from neutral prompts also exhibits greater similarity to those from masculine than from feminine ones.
- Object co-occurrence in images generated from neutral prompts aligns more closely with masculine prompts than with feminine prompts.
- Objects explicitly mentioned in the prompts do not exhibit differences regarding specific gender.
- Objects not explicitly mentioned in the prompts have different possibilities to be generated regarding different genders.

These findings demonstrate that gender bias perpetuates throughout the generating process and manifests across entire images, including areas beyond generated faces. To address this issue, we provide recommendations for both model developers and users to mitigate bias during image generation. Compared to our conference version [19], this work includes the following improvements:

- An extended literature review on gender bias evaluation methods in text-to-image generation.
- Additional details on triplet prompt generation (Section 3.1), image space (Section 4.1.3), and word attention (Section 6.1).
- Expanded experimental results and further discussions in Section 4, Section 5 and Section 6.
- Deeper analysis of the prompt-image dependency, including dependency group presence in images (Section 7.2.1), amount of objects (Section 7.2.2) and group intersection ratio (Section 7.2.3).

## 2. Related Work

### 2.1. Text-to-Image Models

There are three main types of text-to-image generation models: GAN [20,21,22], autoregressive [2,23,24,25,26], and diffusion [1,27,28]. Within diffusion models, Stable Diffusion [1] has emerged as the preferred testbed due to its high-quality generations and open-source nature. As diffusion models rely on cross-attention to connect text and image modalities, it enables the examination of the image generation process at the word level [29]. The cross-attention module assists in tasks such as editing [29,30,31,32,33] and segmentation [34,35,36]. By leveraging this property, we can investigate the relationship between gender and prompt-guided generations.

### 2.2. Social Bias

Text-to-image generation models often reproduce demographic stereotypes tied to gender and race across various factors, including but not limited to occupations [6,7,13,14,15,17,37], adjectives [7,16,38], objects [39], outfits [40], and nationalities [6,41]. Analysis of prompt templates like “a photo of the face of [OCCUPATION]” reveals that certain occupations, such as *software developers*, are predominantly represented as white men, while *housekeepers* tend to be associated with women of color. Additionally, Wolfe et al. [42] showed that models are more inclined to generate sexualized images in response to prompts containing “a [AGE] year-old girl”. Moreover, Zhang et al. [43] argued that unfairness extends to images depicting underrepresented attributes like *wearing glasses*, highlighting the pervasive nature of biases in the generation process. In addition to biases concerning humans, previous studies have explored geographical-level differences in objects [44] and the correctness of cultural context [45,46].

### 2.3. Bias Evaluation

A fundamental aspect in the study of bias is the evaluation protocol. As summarized in Table 1, we compare differences between our method and several previous gender bias evaluation methods in text-to-image generation [4,6,7,13,14,16,39,40,47,48,49,50,51,52,53,54,55,56,57,58]. Most of these approaches rely on prompts that fill attributes (e.g., profession) with a template, leading to constrained scenarios and limited additional details in the prompts. Moreover, these methods evaluate bias on the proxy presentation of the generated images, but do not examine presentations in the generation process. Additionally, these methods mainly focus on people’s attributes, such as the gender of faces, thereby overlooking biases in the generated visual elements as well as the entire image context. Except for the method that exclusively on gender bias evaluation, there are traditional evaluation criteria for text-to-image models measuring image fidelity and text-image alignment with automated metrics [59,60,61,62] or human evaluation [63].

Overall, there is an absence of automated methods for nuanced bias evaluation that conveys bias at the different stages of the generation process. Using free-form prompts, our work proposes a method to uncover prompt-image dependencies, disclosing how objects are generated differently according to gender indicators in the prompt.

## 3. Preliminaries

### 3.1. Triplet Prompt Generation

Let $P_{n}$ be a set of *neutral* prompts, which do not specify the gender of the person. As shown in Figure 1, from these neutral prompts, we generate two counterpart prompt sets, $P_{f}$ and $P_{m}$, as *feminine* and *masculine* prompt sets, respectively. The only difference among these three prompt sets is the gender indicator, while all other words remain unchanged. Our bias evaluation is based on analyzing distinctions between pairs of generated images from the triplet $\left\{P_{n},P_{f},P_{m}\right\}$.

**Figure 2:**
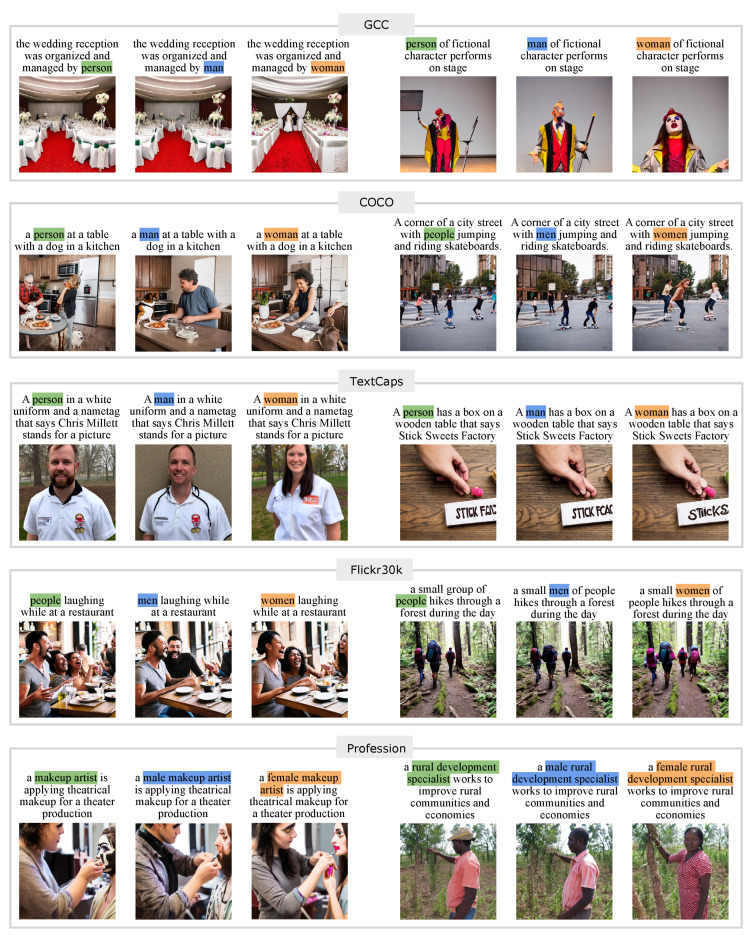
Examples of triplet prompts and the corresponding generated images for each dataset on SD v2.0.

We generate neutral prompts from natural language sentences, consisting of captions from four vision-language datasets (GCC validation set [67], COCO [64], TextCaps [68], and Flickr30k [66]), as well as a profession prompt set generated by ChatGPT 3.5 [18] (accessed on 7 November 2023).From the vision-language datasets, we generate neutral prompts by choosing *neutral captions*. To ensure the neutral prompts do not contain other words that might potentially define the gender of generated people, we set two criteria for the neutral captions: (1) they contain the word *person* or *people*, and (2) they do not include other words (listed in Table 2) that indicate humans (e.g., “The person and a boy are playing badminton” is not a neutral caption). To generate feminine and masculine prompts, we swap *person*/*people* in the neutral captions with the gender indicators *woman*/*women* and *man*/*men*, respectively. For the profession prompt set, we generate neutral prompts with ChatGPT based on professions, such as ecologist or doctor, across 16 topics. Lists of the profession names are presented in Table 3. For example, an ecologist studies the ecosystem in a lush green forest. To create feminine and masculine prompts, we prepend *female*/*male* before the profession (e.g., an *female* ecologist studies the ecosystem in a lush green forest). Examples of triplet prompts and the corresponding generated images for each dataset are shown in Figure 2.

### 3.2. Image Generation

Given prompt *p* as input, Stable Diffusion transforms it into a text embedding t in the *prompt* space using the text encoder. This text embedding is fed into the cross-attention module in UNet [69], which performs the denoising operations from an initial noise $z_{T}$ in the latent space. After *T* denoising steps, the embedding $z_{0}$ in the *denoising* space is obtained. Finally, image *x* in the *image* space is generated from $z_{0}$ by the image decoder. In this work, we evaluate Stable Diffusion models: v1.4 (https://github.com/CompVis/stable-diffusion, accessed on 7 November 2023), v2.0-base (https://huggingface.co/stabilityai/stable-diffusion-2-base, accessed on 7 November 2023), and v2.1-base (https://huggingface.co/stabilityai/stable-diffusion-2-1-base, accessed on 7 November 2023) (denoted as SD v1.4, SD v2.0, and SD v2.1, respectively). The three versions share the same model structure as introduced, but they differ in their text encoders. SD v1.4 uses CLIP ViT-L/14, while SD v2.0 and SD v2.1 use a larger and more transparent encoder, OpenCLIP ViT-H.

Table 4 reports the details of image generation for each dataset. The seed is the same within each triplet, ensuring the same initial noise $z_{T}$. To address data scarcity in GCC and Profession sentences, we produce five images per prompt with five different seeds. In the following, when mentioning a dataset, we are referring to the generated images whose prompts originate from the corresponding dataset.

### 3.3. Gender Bias Definition

The interpretation of gender bias varies across literature, resulting in different work attributing different meanings to the term. In this paper, we define gender bias as follows:

- Within the triplet, images generated from *neutral* prompts consistently display greater similarity to those from either *feminine* or *masculine* prompts.
- Specific objects tend to appear more frequently in the generated images associated with a specific gender.

Whereas objects are not equally distributed in the real world or across cultures, and recognizing that not all disparities regarding genders are inherently problematic (i.e., the association of *dress* with *women* may not be an issue, whereas *kitchen* might), we argue that it is essential to have a methodology for recognizing and quantifying these differences. Our proposed evaluation protocol is not envisaged to identify objects that perpetuate discrimination and gender stereotypes, but to *highlight significant gender disparities*, regardless of whether they are deemed problematic.

## 4. Gender Disparities in Neutral Prompts

**RQ1**  *Do images generated from neutral prompts exhibit greater similarity to those generated from masculine prompts than to images generated from feminine prompts and, if so, why?*

In this section, we address the above research question through the use of representational disparities.

### 4.1. Representational Disparities

**Figure 3:**
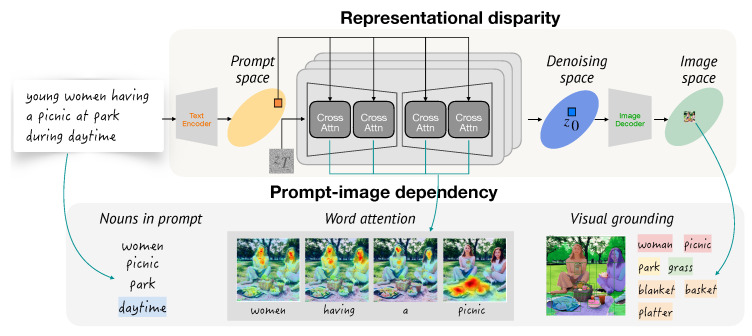
Overview of representational disparities and prompt-image dependency.

We use representational disparities to analyze how images generated by different gender indicators compare with respect to neutral prompts. For a given triplet, the analysis consists on comparing the similarity between *neutral* embeddings and *feminine* and *masculine* embeddings. To measure the extent of gender disparities in the generative process, as shown in Figure 3, we examine the representational disparities throughout the entire generation, tracking embeddings from the prompt space to the denoising space and the image space, offering insights into when bias is introduced.

#### 4.1.1. Prompt Space

The prompt space is defined as the space in which all text embeddings lie. Different points in this space provide different semantics to the following image generation process. To measure the disparity between a pair prompt set P and $P^{\prime }$ in the triplet, we compute cosine similarity as

(1) $$s_{P}\left(P,P^{\prime }\right)=\frac{1}{\left|P\right|}\sum_{p_{i},p_{i}^{\prime }}\cos\left(t,t^{\prime }\right),$$

where |·| is the number of elements in the given set, cos(·,·) gives cosine similarity, the summation is computed over all prompts $p_{i}$ from P and $p_{i}^{\prime }$ from $P^{\prime }$ (subscript *i* is the index of the prompt to clarify $p_{i}$ and $p_{i}^{\prime }$ are corresponding prompts, derived from the same one), and text embeddings t and $t^{\prime }$ correspond to prompts $p_{i}$ and $p_{i}^{\prime }$, respectively.

#### 4.1.2. Denoising Space

The embedding $z_{0}$ after the last denoising process lies in the denoising space. Similarly to the prompt space, we compute cosine similarity as

(2) $$s_{D}\left(P,P^{\prime }\right)=\frac{1}{\left|P\right|}\sum_{p_{i},p_{i}^{\prime }}\cos\left(z_{0},z_{0}^{\prime }\right)$$

where $z_{0}$ and $z_{0}^{\prime }$ are derived from $p_{i}$ and $p_{i}^{\prime }$, respectively.

#### 4.1.3. Image Space

As bias often involves more in the semantics rather than pixel values, we adopt a spectrum of metrics computed from the generated images. To measure image structural differences, we use the average of SSIM scores over all pixels as one of our disparity metrics *SSIM*. Additionally, the ratio of the number of pixels in the contours with higher SSIM scores is used as another disparity metric *Diff. Pix.* To quantify differences in higher-level semantics, we apply latent vectors of pre-trained neural networks, adopting the last fully connected layer of ResNet-50 [70], the image encoder from CLIP ViT-B/32 (https://github.com/openai/CLIP, accessed on 7 November 2023) [71], and the last layer of DINO-s16 [72] following [73], referred to as *ResNet*, *CLIP*, and *DINO*, respectively. For all metrics, we compute the cosine similarity between the latent vectors from image pairs as in Equations (1) and (2). Additionally, we adopt *split-product* [11] using DINO-b8 [72] following the default configuration, computing the maximum cosine similarity among corresponding patches between image pairs.

### 4.2. Results Analysis

By analyzing the representational disparities on (*neutral*, *feminine*), and (*neutral*, *masculine*) pairs, we can provide some answers for **RQ1**.

Results are shown in Table 5. In the image space, regardless of whether considering the entire image holistically (*SSIM*, *Diff. Pix*, *ResNet*, *CLIP*, and *DINO*), or the highest similarity on corresponding patches (*split-product*), images generated from *neutral* prompts consistently demonstrate greater similarity to those from *masculine* prompts. This trend is consistently observed in all datasets and all models.

Tracing back to the prompt space and denoising space to explore where and when gender bias emerges in the generated images, embeddings from *neutral* prompts are closer to the embeddings from *masculine* prompts, both in the prompt space and the denoising space. Although Stable Diffusion models apply different text encoders (OpenCLIP-ViT/H for SD v2.0 and SD v2.1, while CLIP ViT-L/14 for SD v1.4), the same trend is observed across all three models and all datasets. This indicates that gender bias originates from text embedding and perpetuates through the generation process, leading to the disparities observed in the generated images.

## 5. Influence of Gender on Objects

**RQ2**  *Do object occurrences in images significantly vary based on the gender specified in the prompt? If there are differences, do these object occurrences from neutral prompts exhibit greater similarity to those from masculine or feminine prompts?*

The representational disparities reflect the holistic similarity between gender groups, but they do not convey fine-grained differences, i.e., why a certain object appears in the generated image given a gender-specific prompt. In this section, we address **RQ2** by investigating the relationship between gender and the objects in the generated images. To do so, we extract objects with a visual grounding model and study their co-occurrence with each gender.

### 5.1. Detecting Generated Objects

To detect objects in the generated images we use the assembled model Grounded-SAM [74]. Given a generated image, RAM (14M) [75] predicts plausible objects, which are used by Grounded DINO-T [76] to propose bounding boxes around the candidate objects. Then, ViT-H Segment Anything Model (SAM) [77] extracts object regions $m_{o}$ within the bounding box of the object *o*. For each image, a set of object names and a set of regions are obtained.

### 5.2. Evaluation Metrics

Our evaluation protocol involves measuring the differences in object co-occurrences for different genders. Let cnt(o,p) denote the number of occurrences of the object *o* in the image generated from the prompt *p* in the prompt set P. The total number of co-occurrence C(o,P) is given by

(3) $$C\left(o,P\right)=\sum_{p\in P}\mathrm{cnt}\left(o,p\right)$$

With the above definition and a set of triplet prompts, we use the following three methods to evaluate the influence of gender in the generated objects.

(1) Statistical Tests

We use the chi-square test to check whether there are statistical differences in the object co-occurrence among two or three image sets. This test is applicable to the triplet and any pairs in the triplet. If the resulting *p*-value is below 0.05, we interpret significant differences in the object distribution in the pair or triplet.

(2) Co-Occurrence Similarity

We compute the similarity of the co-occurrences of detected objects between two image sets. Formally, let the vector $v_{p}$ denote the object occurrences in the image generated from prompt *p*, and each element in $v_{p}$ is the occurrence cnt(o,p) for the object *o* in the image. Similarly to Equations (1) and (2), we compute cosine similarity on object co-occurrences as

(4) $$s_{O}\left(P,P^{\prime }\right)=\frac{1}{\left|P\right|}\sum_{p_{i},p_{i}^{\prime }}\cos\left(v_{i},v_{i}^{\prime }\right),$$

where prompt sets P and $P^{\prime }$ are in the triplet. $v_{i}$ and $v_{i}^{\prime }$ are derived from prompt $p_{i}$ in P and $p_{i}^{\prime }$ in $P^{\prime }$, respectively. A higher co-occurrence similarity means that objects are detected with the same-level frequency in two image sets, whereas a low similarity means that objects are detected at different rates.

(3) Bias Score

Following [78], we compute the bias score BS(o) for a certain object *o* as

(5) $$\mathrm{BS}\left(o\right)=\frac{C(o,P_{m})}{C\left(o,P_{m}\right)+\frac{|P_{m}|}{|P_{f}|}C\left(o,P_{f}\right)}.$$

BS(o) ranges from 0 to 1, with 1 meaning the object is skewed towards *masculine* prompts and 0 towards *feminine* prompts. If BS(o)=0.5, object *o* does not favor any gender.

### 5.3. Results Analysis

All the *p*-values from chi-square tests among the triplets and pairs are below $10^{-5}$, implying significant differences in the object distributions of each gender across all datasets and models. This shows that according to gender, not only the person in the image may change, but also the objects generated in the image are statistically different.

To investigate whether the object co-occurrences of neutral images exhibit larger similarity to a certain gender image set, we compute co-occurrence similarity on pairs (*neutral, feminine*) and (*neutral, masculine*). Results in Table 6 indicate that object co-occurrences in *neutral* consistently exhibit greater similarity to those in *masculine* prompts than in *feminine* prompts across all datasets and models, corroborating the observations in Section 4. This, again, indicates that prompts that use gender neutral words tend to generated objects that are more commonly generated for masculine prompts than for feminine prompts.

**Figure 4:**
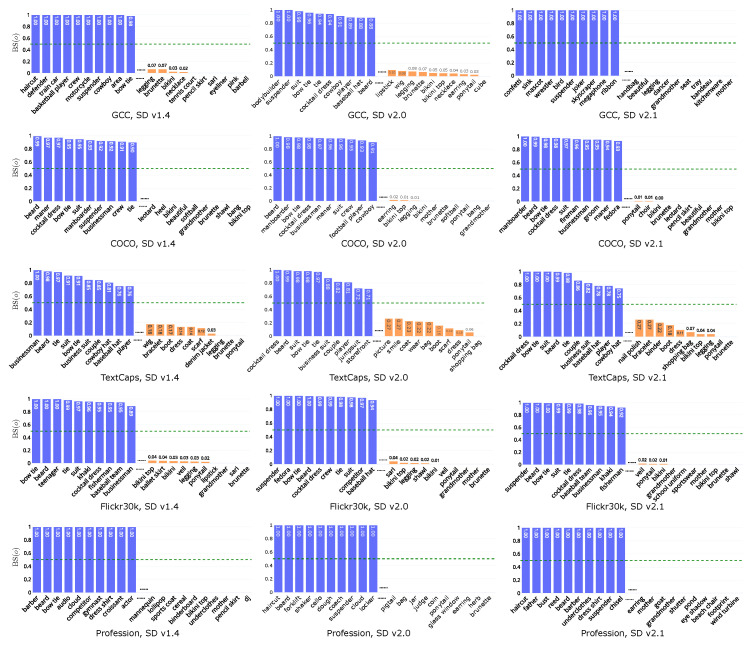
Bias score on all datasets (rows) and models (columns). A high score (blue) indicates the object appears more frequently in masculine, while a low score (orange) suggests the object is more commonly shown in feminine.

Subsequently, we examine specific examples by computing the bias score based on co-occurrence for each object in the generated images. We filter objects if the maximum co-occurrence is less than 10 in GCC, 20 in COCO, TextCaps, and Flickr30k, and 5 in Profession. Results are shown in Figure 4. We can observe that results exhibit a consistent trend across different datasets and models. Take SD v2.0 as an example, notably, clothing and accessory exhibit a high bias: for example, suspender (1 in GCC, Flickr30k, and Profession), suit (GCC, TextCaps, and Flickr30k (0.98), COCO (0.96)), and bow tie (GCC (0.96), COCO and TextCaps (0.98), Flickr30k (1)) lean towards *masculine*, while bikini top (GCC (0.05), COCO (0.01), Flickr30k (0.02)), legging (GCC (0.08), Flickr30k (0.02), COCO (0.01)), and earring (GCC (0.03), COCO (0.02), Profession (0)) lean towards *feminine*. This is not surprising, considering that clothing elements are traditionally gendered. Other than clothing, we find a strong association between family (0.11) and child (0.31) with *feminine* prompts, potentially associating *feminine* with caregiver, while *masculine* prompts exhibit greater alignment with words related to sports such as baseball team (0.91), skateboarder (0.89), and golfer (0.86) (results on Flickr30k, SD v2.0.), a phenomenon that has been previously observed in VQA datasets [79]. Another observation is that *feminine* prompts also have a high association with food, such as salad (0.22), meal (0.25), and cotton candy (0.31) (results on Flickr30k, SD v2.0.). Additionally, results reveal that businessman (COCO, TextCaps, and Flickr30k on SD v1.4, COCO on SD v2.0, COCO and Flickr30k on SD v2.1) tends to be skewed towards *masculine* whereas kitchenware (GCC, SD v2.1) tends to be associated with *feminine* prompts.

## 6. Gender in Prompt-Image Dependencies

**RQ3**  *Does the gender in the input prompt influence the prompt-image dependencies in Stable Diffusion, and if so, which prompt-image dependencies are more predisposed to be affected?* 

To answer this question, we need to know not only which objects are generated for each gender, but also how each object is generated in the diffusion process. To do so, we propose to classify objects into prompt-image dependency groups according to their relationship with the input prompt and the generated image. First, we conduct an *extended object extraction* by detecting not only the objects in the generated image, as in Section 5, but other objects also involved in the generative process. Then, we classify each object according to five *prompt-image dependency groups*, which allows us to study how gender influences objects according to their generative process.

### 6.1. Extended Object Extraction

To detect extended objects involved in the generative process, we conduct three extraction processes (see the example in “Prompt-image dependency” part of Figure 3).

  **(1) Nouns in prompt.**

Prompts, designed by users, are a direct cue of what they wish to see in the generated image. The generated image, on the other hand, is required to be faithful to the prompt. The first extraction process targets nouns within the prompt, recognizing their importance in directly shaping the occurrence of objects in the generated image. For each prompt, we obtain a noun set including all lemmatized nouns *n* in the prompt by using NLTK [80].

  **(2) Word attention.**

Verifying whether objects in the noun set are faithfully generated in the image is demanding, as it requires locating the region that the noun guides. Fortunately, cross-attention has proven to be effective in exploring the word guidance during the generation process [29,34]. Our second extraction process is the word attention masks generated by the cross-attention module via DAAM [34]. In detail, let P be a matrix whose column *n* is the word embedding corresponding to the word *n* in *p*, and $H(z_{t})$ be a feature map of a certain block of Stable Diffusion’s UNet for latent embedding $z_{t}$ in the *t*-th denoising step. Cross-attention between P and $H(z_{t})$ is given by

(6) $$\begin{array}{c} A_{t}=\mathrm{softmax}\left(\frac{QK^{⊤}}{\sqrt{d}}\right), \end{array}$$

where Q and K are the query and key matrices given using linear layers $W_{Q}$ and $W_{K}$ as $Q=W_{Q}H\left(z_{t}\right)$ and $K=W_{K}P$, whose output dimensionality is *d* (the index *t* for denoising step is omitted for simplicity). The heart of DAAM is $A_{t}$, of which column *n* is the attention map from word *n* to each spatial position of feature map $H(z_{t})$. We aggregate the attention maps over UNet blocks, multiple attention heads, and denoising steps. Let $\alpha_{n}$ denote the attention map, reshaped and resized to the same size as the corresponding generated image *x*, normalized to [0,1]. For each word, we first compute the normalized attention map, where a higher value indicates that the pixel is more associated with the word. Then, we binarize the attention map with a threshold θ to obtain a set of masks $a_{n}$, responding to the region of an object specified by the word *n*. In each prompt, we obtain a mask set containing the mask $a_{n}$ for each word *n*. We set threshold θ as 0.35.

  **(3) Visual grounding.**

Nouns and the corresponding object regions cover only a small subset of objects in the generated image; there should be many other objects that are not explicitly described in the prompt, but are still included in the image to complete the scene. We aim to enumerate as many objects as possible for comprehensive object-level analysis. To spot regions of arbitrary objects, the last extraction process is the same visual grounding process as in Section 5.

### 6.2. Prompt-Image Dependency Groups

Next, we classify each detected object according to its generative process. On the one hand, the generated image should align with its prompt, which can be verified using the noun set and the mask set. On the other hand, the image may have other visual elements beyond the prompt, listed in the object set and the object region set. To define prompt-image dependency groups, we consider the dependency among objects, the noun set, and the mask set based on its membership.

**Definition** **1** (Explicitly). *If the object o is in the noun set, it is explicitly described in the prompt.*

**Definition** **2** (Guided). *If object region $m_{o}$ sufficiently overlaps with at least one mask in the mask set, the object o is guided by cross-attention between the prompt and the image. Sufficiency is determined by the coverage of object region $m_{o}$ by the mask a,*

(7) $$\mathrm{coverage}\left(m_{o},a\right)=\frac{|m_{o}\cap a|}{|m_{o}|},$$

*where |·| is the number of pixels. Thus, if $\mathrm{coverage}(m_{o},a)$ is larger than a certain threshold σ, the object region $m_{o}$ sufficiently overlaps with the mask a. We set different values for threshold σ for words referring to humans (people, person, woman, women, man, men) versus objects. The reason is that for human words, the generated people in the images can still be considered as guided by those words, even when the coverage (Equation* (7) *of word attention and visual grounding is relatively low. For example, word attention on human words may focus only on the face, while the visual grounding covers the whole body. Since these partial overlap cases are common for human words, we set a lower threshold of σ=0.25 when both the detected object and word refer to humans. For all other cases, a higher threshold of σ=0.7 is used.*

**Figure 5:**
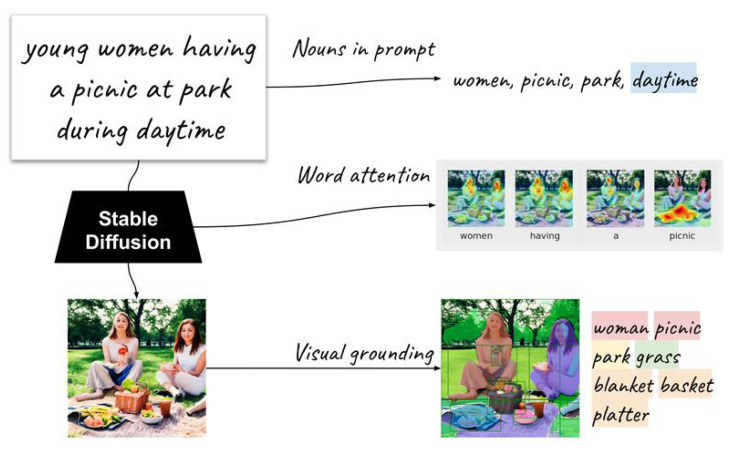

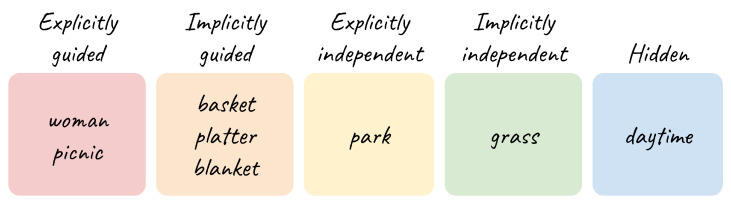
Prompt-image dependency groups.

With these definitions, we cluster objects in the object set into five groups, as illustrated in Figure 5 with the example prompt young women having a picnic at the park during daytime.

**Explicitly guided**.  The object is *explicitly* mentioned in the prompt, and *guided* by cross-attention. Faithful image generation may require each noun to be associated with the corresponding object.
**Implicitly guided**.  The object is *not explicitly* mentioned in the prompt, but *guided* by cross-attention. The object may be strongly associated with or pertain to a certain noun in the noun set, e.g., the object basket for the noun picnic.
**Explicitly independent**.  The object is *explicitly* mentioned in the prompt, but *not guided* by cross-attention. e.g., park.
**Implicitly independent**.  The object is *not explicitly* mentioned in the prompt, and *not guided* by cross-attention. The object is generated solely based on contextual cues, e.g., grass.
**Hidden**.  The noun has no association with objects in the object set, i.e., the noun is *not included* in the images, e.g., daytime.

Figure 5 illustrates the object extraction processes and the resulting dependency groups. Dependency groups are important as they depict if an object tends to appear, for example, in relation to the prompt (*explicitly guided*) or just for filling the scene (*implicitly independent*). Together with the gender-specific sets of prompts, they vividly provide essential insights into how an image generation model behaves for different genders.

### 6.3. Result Analysis

We denote co-occurrence $C_{g}\left(o,P\right)$ as the number of occurrences of object *o* in each dependency group *g*. To clarify, given that there are nouns included in the hidden group, the computation of occurrence should be adjusted from $C_{g}\left(o,P\right)$ to $C_{g}\left(n,P\right)$ for *n* in the hidden group.

#### 6.3.1. Objects in Dependency Groups

**Figure 6:**
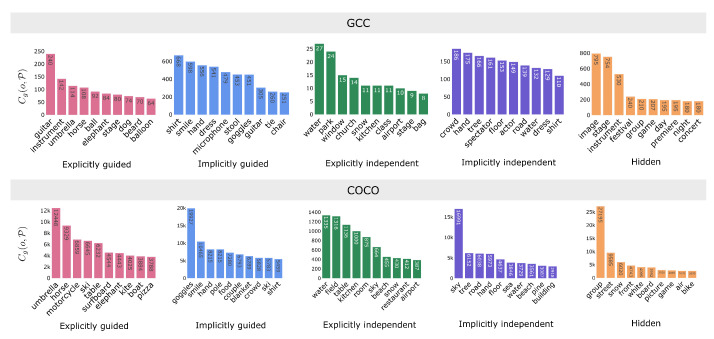

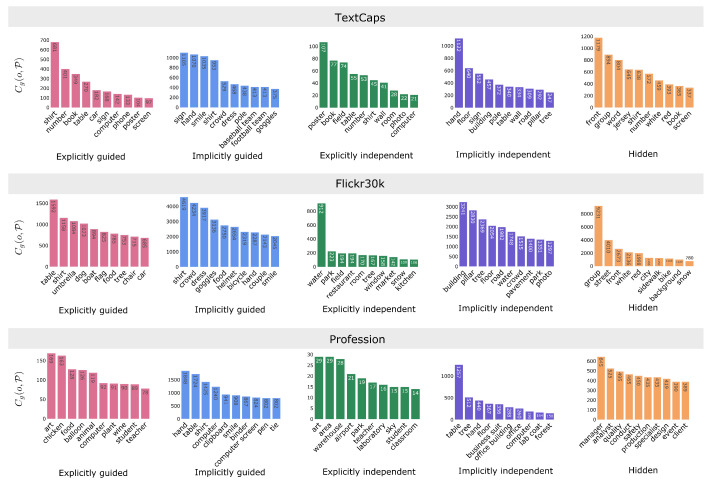
The occurrence $C_{g}\left(o,P\right)$ of object *o* in images generated from P on each dependency group for each dataset (SD v2.0).

To answer RQ3, we first investigate objects in the prompt-image dependency groups, aiming to identify which types of objects are generated under the influence of the prompt, the cross-attention, or the context of the generated image. Figure 6 shows the prevalent objects within each dependency group across all datasets on SD v2.0 (to focus on the differences between generated objects, we remove individuals (person, people, women, woman, men, man, female, male, girl, boy)). Although the specific generated objects align with the prompt’s domain, and their frequencies may vary across datasets, we observe consistent trends.

Objects in the *explicitly guided* group include animals and tangible items commonly encountered in daily life, such as umbrella and table. The *implicitly guided* group contains objects surrounding human beings, such as clothing and personal belongings like shirt and goggles. The *explicitly independent* group comprises words related to the surrounding environment, such as kitchen or restaurant. Objects in the *implicitly independent* group are typically part of the background that can be detected, like tree and road, along with attire accompanying individuals. Lastly, the *hidden* group comprises words challenging to detect in images, such as game and air.

#### 6.3.2. Gender and Dependency Groups

Next, we investigate the relationship between gender and the objects in each prompt-image dependency group. To discern whether object differences are statistically significant, we conduct chi-square tests on the object co-occurrence for each dependency group. Table 7, Table 8 and Table 9 present chi-square test results on dependency groups in each dataset on SD v1.4, SD v2.0, and SD v2.1, respectively. The same setting is applied as in Section 5. It is observed that, except for COCO, other datasets have a similar distribution in the *explicitly guided* and *explicitly independent* groups. Moreover, all datasets show significant differences in the *implicitly guided* and *implicitly independent* groups. Thus, we subsequently investigate objects in these two groups.

While we find significant differences (*p*-value < 0.05) across all datasets in the *implicitly guided* and *implicitly independent* groups, we do not find significant differences in most datasets in the *explicitly guided*, *explicitly independent*, and *hidden* groups. This suggests that while Stable Diffusion may consistently generate the nouns explicitly mentioned in the prompt, it may rely on gender cues for generating elements that are not specified in the prompt, such as the background and surroundings of the individuals.

**Figure 7:**
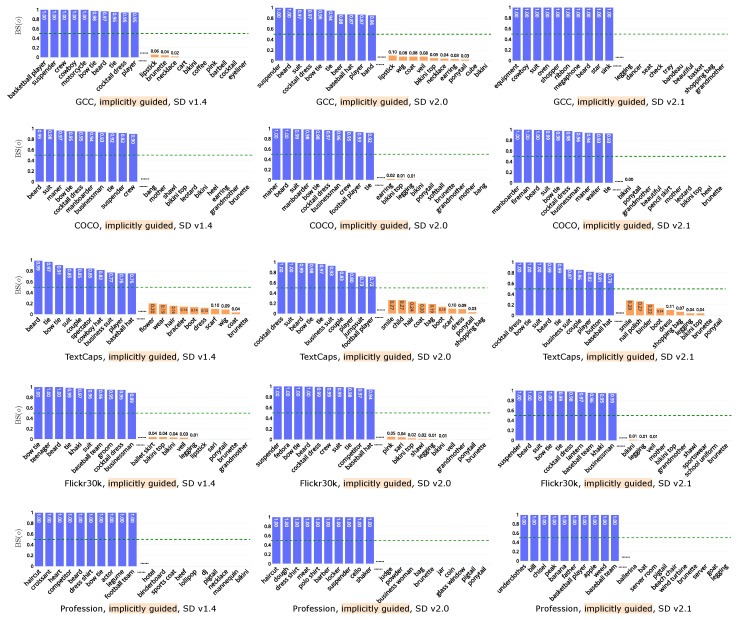
Bias score on *implicitly guided* on the datasets (rows) and models (columns).

To further explore the text-image dependencies and their correlation with gender, we calculate the bias score based on object co-occurrence in *implicitly guided* and the *implicitly independent* groups, both of which exhibit statistically significant differences. Figure 7 shows the top-10 objects skewed toward *masculine* and *feminine* in *implicitly guided* on all datasets and models. We analyze results on SD v1.4 as examples. For the *implicitly guided* group, we observe high bias scores for clothing items, such as cocktail dress (GCC, COCO, and Flickr30k (0.95)), suit (COCO (0.98), TextCaps (0.85)), and bow tie (GCC (0.98), COCO (0.95), TextCaps (0.91), Flickr30k and Profession (1)) for *masculine*, and bikini (GCC, COCO, and Profession (0), Flickr30k (0.04)), dress (COCO (0.12)) and boot (TextCaps (0.14)) for *feminine*, aligning with observations in a previous work [40]. Another prominent observation, consistent with the findings in RQ2, is the strong association of child (0.27) with *feminine*, and *masculine* with sports-related terms such as player (0.8) and football player (0.72) (results on TextCaps, SD v2.0.). Similar gendered associations are observed across different datasets and models.

**Figure 8:**
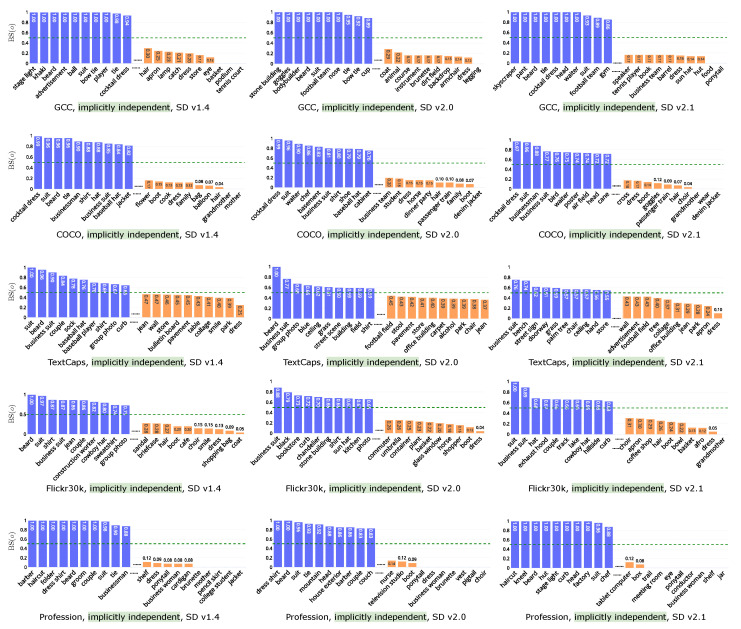
Bias score on *implicitly independent* on the datasets (rows) and models (columns).

Figure 8 shows the bias scores in *implicitly independent* across all datasets and models. While places and surroundings are the majority (as discussed in Section 6.3.1), clothing associated with individuals in the *implicitly independent* group may exhibit higher or lower bias scores. Given the similar trend in clothing between *implicitly guided* and *implicitly independent*, we focus on surroundings and other items in the latter group. As the specific environments generated are influenced by the semantics of the text, we conduct analysis based on datasets. In COCO, results show that basement and cabinet are more prone to appear in *masculine*, while dinner party, and passenger train are inclined to be generated in *feminine*. In TextCaps, grass, building, and field are skewed toward *masculine*, while park, carpet, and store are skewed toward *feminine*. Taking GCC on SD v2.0 as an example, sports-related items such as bodybuilder (1) and football team (1) are again skewed toward *masculine*, while instrument (0.17) and apron (0.33) are more aligned with *feminine*. Additionally, there are also disparities related to backgrounds, such as backdrop (0.15) and dirt field (0.17) for *feminine*, and stone building (1) and tennis court (0.63) for *masculine*. Furthermore, certain words consistently align with feminine across datasets and models, such as smile (TextCaps and Flickr30k on SD v1.4) and flower (COCO on SD v1.4).

## 7. Additional Experiments

To further evaluate our protocol, we conduct intra-prompt evaluation and human evaluation.

### 7.1. Intra-Prompt Evaluation

To eliminate the influence of randomness, we investigate the research questions using images generated from the same triplet prompts. We generate a total of 3000 images on 1000 seeds with SD v2.0, from triplet prompts derived from a caption in GCC: “*person looks at the falling balloons at the conclusion*”. We use the same settings as conducted in the experiments above.

For RQ1, the representational disparities in Table 10 show that *neutral* is consistently closer to *masculine* in each space. For RQ2, the chi-square tests on the object occurrences among the triplets and every pair within the triplets, *p*-value is consistently less than $10^{-5}$, indicating statistically significant differences. For RQ3, the chi-square tests also reveal significant differences in the groups *implicitly guided* and *implicitly independent* ($p<10^{-5}$). However, we do not apply chi-square tests to *explicitly guided*, *explicitly independent*, and *hidden*, as the numbers of objects in these groups are less than 5. The co-occurrence similarity $s_{O}\left(P_{n},P_{f}\right)$ between the neutral and feminine is 0.733, while the similarity $s_{O}\left(P_{n},P_{m}\right)$ between the neutral and masculine is 0.773. This indicates that the object co-occurrences in images generated from *neutral* prompts are closer to those from *masculine* prompts than *feminine* prompts. These findings correspond to the above results.

### 7.2. Dependency Groups Analysis

Taking Stable Diffusion v2.0 as an example, we scrutinize the dependency groups deeper to discover the underlying connections between groups and objects.

#### 7.2.1. Dependency Group Presence in Images

To assess the presence of dependency groups in images, we compute the percentage of the images containing dependency groups over the total number of images in each dataset. The results are presented in Table 11. For example, in the GCC dataset, 64.48% images contain at least one object in the *explicitly guided* group. Similarly, in other datasets, over 60% of images have objects in the *explicitly guided*, except for the Profession set, where the proportion is only 15%. This disparity may be due to the specialized terminology in the Profession set, potentially reducing the chance of being detected by the visual grounding model. Conversely, only around 10% or fewer images include objects in the *explicitly independent* group. Given that most objects in this group represent the surrounding environment, objects in *explicitly independent* may occur when the prompt contains words indicating the surrounding environment (e.g., park, kitchen).

Moreover, a similar trend is observed across all datasets, where most images contain objects from the *implicitly guided*, *implicitly independent*, and *hidden* group. This indicates that text-to-image models generate auxiliary objects to fill in both the areas guided by the prompt and those independent from it. We posit that the high proportion of *hidden* group may be due to the abstract words that are challenging to detect and to the mismatch in synonyms. For instance, the visual grounding model may struggle to identify people as professions in the Profession set.

#### 7.2.2. Amount of Objects

Next, we investigate the amount of individual objects in each dependency group and nouns in prompts. The results for each dataset are shown in Table 12. Supporting the findings in Table 11, objects in the *explicitly guided* and *explicitly independent* constitute only a small portion of the nouns in the prompts. Additionally, despite not being mentioned in the prompt, *implicitly guided* and *implicitly independent* groups contain more objects than *explicitly* groups present in the image. This suggests that these two *implicitly* groups are worth further exploration for a comprehensive understanding of the image generation process.

#### 7.2.3. Group Intersection Ratio

To uncover the common patterns and potential connections among the groups, we report the intersection ratio of individual objects among dependency groups and nouns in the prompts in Table 13. The ratio in each cell is computed from the intersection of two groups over the group in the stub column. For example, in the GCC dataset, 76.77% of objects in the *explicitly guided* are also included in the *implicitly guided* group.

Similar trends are observed across all datasets. Thus, we take GCC as an example. We observe that many objects in the *explicitly* groups are also found in the *implicitly* groups. This suggests that these objects are more likely to be generated and detected, even if they are not explicitly mentioned in the prompts. Additionally, most objects in the *explicitly* groups are also included in the *hidden* group. This may due to potential mismatches in synonyms, and there may be cases where the objects are not generated or detected.

On the other hand, within the *implicitly* groups, only a small fraction (about 10%) of objects are also present in the *explicitly* group. This indicates that these objects are used to fill the scene, but are not likely to be explicitly mentioned in the prompt. For example, with a prompt like “a person is walking along the street”, it is common if the generated image contains pavement. However, pavement might not be explicitly mentioned unless the scene specifically relates to it, such as “a person is crossing the pavement”.

### 7.3. Human Evaluation

To evaluate the reliability of the visual grounding model, we randomly select 100 generated images from SD v2.0 along with the nouns from the corresponding prompts and conduct a human evaluation to determine whether the nouns are present in the images. The 100 prompts contain 346 nouns, from which 227 (65.61%) are correctly identified both by humans and the automated vision grounding. Out of the remaining 119 nouns, only 8 nouns are detected by the model but not observed by humans. These nouns are frisbee (2), women (1), people (1), kite (1), scooters (1), tennis (1), and speaker (1). For the nouns not detected by the model but identified by humans, the most frequent ones are woman (10), street (7), people (6), and snowy (4). The absence of the noun street in the model’s detection might be attributed to the strict alignment between nouns and objects. Even if the model successfully identifies street scene, the specific noun street might be placed in one of the *implicitly guided*, *implicitly independent*, or *hidden* groups. These results indicate that the visual grounding model has reasonable accuracy in detecting nouns appearing in the generated images, though there is still room for improvement on abstract nouns and scene-level nouns.

## 8. Recommendations

Our methodology revealed significant disparities in the objects generated by three Stable Diffusion models according to the gender in the input prompt. While these discrepancies may seem harmless, they can potentially reinforce gender stereotypes. With this in mind, we propose a series of suggested practices aimed at mitigating these concerns, both for model developers and for users:

### 8.1. Model Developers

#### 8.1.1. Debias Text Embeddings

We have identified that gender bias originates in the text embedding, with *neutral* prompts consistently being more similar to *masculine* prompts than to *feminine* prompts, which propagates through the entire generation process. Given the documented presence of gender bias in CLIP [42,81,82,83], it comes as no surprise that text-to-image generation models relying on CLIP also exhibit such biases. The first mitigation technique should focus on debiasing the text embedding space, aiming for more equitable representations.

#### 8.1.2. Identify Problematic Representations

While some associations of certain objects with specific genders may not immediately raise concerns, others could potentially do so. Therefore, researchers must meticulously assess these associations, taking into account the cultural context in each instance. It is crucial to examine the co-occurrence of objects across genders and check whether neutral prompts tend to exhibit a preference toward a particular gender.

#### 8.1.3. Investigate Modules That Complete the Scene

Significant differences were observed in the *implicitly* generated objects, underscoring the need to investigate how the model completes the scene. Future research could explore other modules, probing fine-grained control over the regions not guided by the input.

### 8.2. Users

#### 8.2.1. Explicitly Specify Objects

Our results showed that there are no significant differences in the objects explicitly mentioned in the input prompts concerning gender. This suggests that Stable Diffusion models can adhere to the simple instructions in the prompt regardless of gender. Therefore, expanding the number of objects in the input could offer greater control over broader guided regions and potentially lead to the generation of images with less gender disparity.

#### 8.2.2. Explicitly Specify Gender

Considering that *neutral* prompts consistently produced images more similar to those from *masculine* prompts, we advise refraining from using neutral prompts if targeting a balanced distribution across genders. Instead, using prompts with specified gender indicators may be more reliable.

## 9. Limitations

We acknowledge that our proposed evaluation protocol has limitations, and we emphasize them here for transparency and to inspire the community to propose enhancements in future studies. Firstly, our evaluation protocol focuses on binary genders, neglecting to evaluate gender from a broader spectrum perspective. To enhance inclusivity, future research could extend the analysis to encompass a more diverse range of genders. Secondly, our protocol relies on a stringent alignment between nouns and objects, assuming their identity after lemmatization, which may overlook variations and synonyms. Thirdly, the objects segmented in visual grounding may encounter errors, possibly perpetuating issues in the classified groups. Additionally, if gender bias exists in the visual grounding model, where certain objects may be more challenging to detect in specific genders, this bias could transfer to the final results. Additionally, when the object comprises more than one word (e.g., “picnic basket”), each noun in the phrase has its own word attention rather than being considered as a single entity. Last but not least, our study only examines the presence of objects not differentiating with distinct attributes, such as color or shape.

## 10. Conclusions

We introduced an automated evaluation protocol to study gender bias in image generation by probing the internal components of Stable Diffusion models. We investigated both representational disparities and prompt-image dependencies to uncover the origin of bias and how it manipulated image generation. Through the generation of free-form triplet prompts with only gender indicators differing, our findings indicate the following:

1. Prompts that use *neutral* words to refer to people (a person in a park) consistently yield images more similar to the ones generated from prompts with *masculine* words (a man in a park) than from prompts with *feminine* words (a woman in a park).
2. There are statistically significant differences in the type of objects generated in the image based on the gender indicators in the prompt.
3. The frequency of objects generated explicitly from prompts exhibit similar behavior for different genders.
4. Objects not explicitly mentioned in the prompt exhibit significant differences for each gender.
5. We particularly observed significant statistical disparities in generated objects based on gender in items related to clothing and traditional gender roles such as sports, which are highly skewed towards images generated from *masculine* prompts, and food, which are skewed towards images generated from *feminine* prompts.

Based on these observations, we provided recommendations for developers and users to reduce such representational disparities and gender bias in the generated images. We hope these insights contribute to underscoring the nuanced dynamics of gender bias in image generation, offering a new and valuable perspective to the growing body of research on this topic.

## Figures and Tables

**Table 1:** Gender bias evaluation methods in text-to-image generation. We compare with previous methods on input (prompt type, prompt variation), evaluation space (prompt, denoising, image), and bias (subject of bias). “Prompt variation” refers to how prompts vary in attributes (e.g., profession) while keeping other words unchanged when the prompts are template-based. If prompts are from caption datasets, the specific dataset names are presented. In terms of the “subject of bias”, *gender* means the gender of generated faces, while *performance* contains generation performance metrics such as text-to-image alignment and image quality.

Method	Input		Evaluation Space			Bias
	Prompt Type	Prompt Variation	Prompt	Denoising	Image	Subject of Bias
[7]	Template	Identity, Profession	-	-	✓	Gender
[47]	Free-form	Objects	-	-	✓	Performance
[48]	Template	-	-	-	✓	Gender
[49]—Fairness	Free-form	COCO [64]	-	-	✓	Performance
[49]—Bias	Template	Adjective, Profession	-	-	✓	Gender
[13]	Template	Profession	-	-	✓	Gender, Attire
[6]	Template	Profession	-	-	✓	Gender
[14]—Profession	Template	Profession	✓	-	✓	Gender
[14]—Science/Career	Template	Science, Career	-	-	✓	Gender
[50]	Free-form	Creative prompts, Diffusion DB [65]	-	-	✓	Concept
[40]	Template	Attire, Activity	-	-	✓	Attire
[16]	Template	Adjective, Profession	-	-	✓	Gender
[16]—Expanded	Template	Profession	-	-	✓	Gender, Performance
[51]	Template	Adjective, Profession, Multilingualism	-	-	✓	Gender
[52]	Free-form	Profession	-	-	✓	Gender
[53]	Template	Profession, Social relation, Adjective	-	-	✓	Gender
[54]	Template	Action, Appearances	-	-	✓	Gender
[55]	Template	Two professions	-	-	✓	Gender
[56]	Template	Activity, Object, Adjective, Profession	-	-	✓	Gender
[57]	Free-form	Flickr30k [66], COCO [64]	-	-	✓	Gender
[39]	Template	-	-	-	✓	Object
[4]	Free-form	PHASE [4]	-	-	✓	Safety
[58]	Template	Profession, Sports, Objects, Scene	✓	-	✓	Gender
Ours	Free-form	GCC [67], COCO [64], TextCaps [68], Flickr30k [66], Profession	✓	✓	✓	Layout, Objects

**Table 2:** Words that indicate humans.

Type	Word
Gender	woman, female, lady, mother, girl, aunt, wife, actress, princess, waitress, sister, queen, pregnant, daughter, she, her, hers, herself, bride, mom, queen, man, male, father, gentleman, boy, uncle, husband, actor, prince, waiter, son, brother, guy, emperor, dude, cowboy, he, his, him, himself, groom, dad, king
Geography	American, Asian, African, Indian, Latino
Others	commander, officer, cheerleader, couple, player, magician, model, entertainer, astronaut, artist, student, politician, family, guest, driver, friend, journalist, relative, hunter, tourist, chief, staff, soldier, civilian, author, prayer, pitcher, singer, kid, groomsman, bridemaid, ceo, customer, dancer, photographer, teenage, child, u, me, I, leader, crew, athlete, celebrity, priest, designer, hiker, footballer, hero, victim, manager, Mr, member, partner, myself, writer

**Table 3:** Profession names in the Profession set.

Topic	Profession Name
Science	Botanist, Geologist, Oceanographer, Astronomer, Meteorologist, Chemist, Physicist, Geneticist, Archaeologist, Biostatistician, Marine Biologist, Quantum Physicist, Seismologist, Ecologist, Geophysicist, Epidemiologist, Materials Scientist, Neuroscientist, Volcanologist, Zoologist
Art	Street Artist, Songwriter, Calligrapher, Art Appraiser, Tattoo Artist, Mural Artist, Writer, Illustrator, Film Director, Ceramic Artist, Curator, Makeup Artist, Graffiti Artist, Furniture Designer, Cartoonist, Sculptor, Fashion Designer, Glassblower, Landscape Painter, Storyboard Artist
Sports	Athlete, Gymnast, Swimmer, Runner, Cyclist, Skier, Diver, Wrestler, Boxer, Surfer, Coach, Fitness Instructor, Sports Photographer, Referee, Sports Agent, Soccer Player, Tennis Coach, Yoga Instructor, Martial Arts Instructor, Golf Caddy
Celebrations	Wedding Planner, Party Decorator, Event Caterer, Balloon Artist, Fireworks Technician, Event DJ, Wedding Officiant, Event Photographer, Costume Designer, Event Coordinator, Cake Decorator, Floral Designer, Lighting Technician, Ice Sculptor, Musician, Face Painter, Magician, Pyrotechnician, Caricature Artist, Audiovisual Technician
Education	School Principal, Librarian, Academic Advisor, Teaching Assistant, School Psychologist, Early Childhood Educator, Curriculum Developer, Educational Technologist, Special Education Teacher, School Counselor, Online Instructor, Music Teacher, Art Teacher, Mathematics Teacher, Science Teacher, History Teacher, Language Teacher, Physical Education Teacher, College Professor, Career Counselor
Healthcare	Nurse, Doctor, Therapist, Surgeon, Pharmacist, Midwife, Paramedic, Psychologist, Radiologist, Dentist, Orthopedic Surgeon, Oncologist, Pediatrician, Anesthesiologist, Dermatologist, Neurologist, Cardiologist, Chiropractor, Veterinarian, Respiratory Therapist
Technology	Data Analyst, Information Security Analyst, AI Ethics Researcher, Virtual Reality Developer, Quantum Computing Researcher, Ethical Hacker, Robotics Engineer, Software Developer, Database Administrator, Network Engineer, Machine Learning Engineer, Cybersecurity Consultant, Web Developer, Cloud Architect, Digital Marketing Specialist, IT Support Specialist, Game Developer, UI Designer, Biomedical Engineer, Tech Startup
Business and Finance	Business Analyst, Tax Consultant, Financial Planner, Corporate Risk Manager, Actuary, Import-Export Specialist, Accountant, Investment Analyst, Operations Manager, Management Trainer, Small Business Consultant, Financial Auditor, Financial Controller, Human Resources Manager, Marketing Manager, Real Estate Agent, Supply Chain Manager, Chief Financial Officer, Economist, Chief Executive Officer
Government and Public Service	Diplomatic Services Officer, Social Services Worker, Public Policy Analyst, Environmental Health Inspector, Fire Marshal, Immigration Officer, Park Ranger, Community Organizer, Census Bureau Statistician, Emergency Management Director, Social Worker, Police Officer, Public Health Inspector, Environmental Scientist, City Planner, Legislative Aide, Judge, Foreign Service Officer, Conservation Officer, Civil Servant
Agriculture and Farming	Organic Farming Consultant, Beekeeper, Nutritionist, Agricultural Inspector, Poultry Farmer, Soil Conservationist, Aquaculture Technician, Agricultural Economist, Irrigation Specialist, Farm Equipment Mechanic, Livestock Rancher, Horticulturist, Viticulturist, Dairy Farmer, Agricultural Researcher, Fishery Manager, Rural Development Specialist, Animal Breeder, Greenhouse Manager, Sustainable Agriculture Advocate
Environmental	Wildlife Biologist, Environmental Educator, Green Building Architect, Environmental Geologist, Air Quality Specialist, Water Quality Analyst, Forest Ranger, Marine Ecologist, Climate Change Analyst, Conservation Biologist, Park Naturalist, Wetland Scientist, Renewable Energy Specialist, Sustainability Consultant, Eco-Tourism Guide, Environmental Impact Analyst, Land Use Planner, Soil Scientist, Environmental Policy Analyst, Recycling Coordinator
Travel and Hospitality	Travel Agent, Tour Guide, Hotel Manager, Flight Attendant, Cruise Ship Staff, Concierge, Restaurant Manager, Sommelier, Travel Blogger, Amusement Park Entertainer, Culinary Tour Guide, Hotel Concierge, Resort Manager, Airport Operations Manager, Tourism Marketing Specialist, Hospitality Sales Manager, Bed and Breakfast Owner, Cabin Crew Member, Theme Park Performer, Hostel Manager
Media and Journalism	War Correspondent, Documentary Filmmaker, Social Media Influencer, Radio Show Host, Film Critic, Multimedia Journalist, Travel Photographer, Sports Anchor, News Producer, Investigative Journalist, Foreign Correspondent, Photojournalist, Columnist, Podcast Host, Public Relations Specialist, Media Critic, Weather Forecaster, Press Secretary, News Editor, TV News Reporter
Law and Legal	Lawyer, Intellectual Property Attorney, Criminal Psychologist, Legal Ethicist, Court Clerk, Arbitrator, Paralegal, Legal Secretary, Legal Consultant, Immigration Attorney, Family Law Mediator, Legal Aid Attorney, Bankruptcy Attorney, Legal Translator, Corporate Counsel, Tax Attorney, Civil Litigation Attorney, Legal Auditor, Criminal Defense Attorney, Judicial Law Clerk
Manufacturing and Industry	Quality Assurance Manager, Industrial Hygienist, Production Scheduler, CNC Machinist, Factory Inspector, Metallurgical Engineer, Assembly Line Worker, Process Improvement Specialist, Materials Handler, Manufacturing Engineer, Welder, Packaging Technician, Facilities Manager, Maintenance Technician, Logistics Coordinator, Lean Manufacturing Specialist, Safety Coordinator, Inventory Control Analyst, Machine Operator, Operations Supervisor
Culinary and Food Services	Food Safety Inspector, Mixologist, Chef, Brewery Master, Baker, Restaurant Critic, Sommelier, Food Scientist, Caterer, Nutritionist, Butcher, Pastry Chef, Culinary Instructor, Wine Taster, Gourmet Food Store Owner, Food Stylist, Coffee Roaster, Line Cook, Chocolatier, Food Truck Owner

**Table 4:** Number of generated triplets, prompts, and images for each dataset.

Data	Triplets	Prompts	Seeds	Images
GCC (val)	418	1254	5	6270
COCO	51,219	153,657	1	153,657
TextCaps	4041	12,123	1	12,123
Flickr30k	16,507	49,521	1	49,521
Profession	811	2433	5	12,165

**Table 5:** Representational disparities between neutral, feminine, and masculine prompts in the three spaces on Stable Diffusion models.

Pairs		Prompt	Denoising	Image					
		t	$z_{0}$	*SSIM* ↑	*Diff. Pix.* ↓	*ResNet* ↑	*CLIP* ↑	*DINO* ↑	*Split-Product* ↑
SD v1.4									
GCC									
	(neu, fem)	0.909	0.770	0.516	42.61	0.848	0.794	0.543	0.956
	(neu, mas)	0.931	0.798	0.543	39.34	0.859	0.808	0.576	0.961
COCO									
	(neu, fem)	0.920	0.778	0.568	38.558	0.866	0.8584	0.564	0.957
	(neu, mas)	0.942	0.796	0.592	35.671	0.873	0.8580	0.591	0.959
TextCaps									
	(neu, fem)	0.931	0.747	0.461	46.873	0.853	0.773	0.530	0.952
	(neu, mas)	0.948	0.768	0.487	43.599	0.862	0.786	0.555	0.954
Flickr30k									
	(neu, fem)	0.913	0.792	0.492	44.010	0.858	0.830	0.563	0.959
	(neu, mas)	0.931	0.804	0.518	41.105	0.865	0.828	0.587	0.960
Profession									
	(neu, fem)	0.854	0.765	0.487	45.006	0.831	0.830	0.528	0.948
	(neu, mas)	0.862	0.783	0.508	42.528	0.843	0.846	0.555	0.952
SD v2.0									
GCC									
	(neu, fem)	0.980	0.767	0.543	39.00	0.847	0.797	0.545	0.957
	(neu, mas)	0.982	0.790	0.571	35.82	0.864	0.817	0.581	0.963
COCO									
	(neu, fem)	0.984	0.793	0.603	34.10	0.881	0.861	0.595	0.9645
	(neu, mas)	0.985	0.805	0.616	32.50	0.887	0.859	0.609	0.9647
TextCaps									
	(neu, fem)	0.9846	0.745	0.502	41.41	0.861	0.771	0.536	0.958
	(neu, mas)	0.9854	0.767	0.530	37.41	0.874	0.791	0.570	0.962
Flickr30k									
	(neu, fem)	0.982	0.801	0.541	38.42	0.871	0.833	0.584	0.9685
	(neu, mas)	0.983	0.809	0.559	36.02	0.874	0.826	0.601	0.9686
Profession									
	(neu, fem)	0.85784	0.766	0.511	42.41	0.839	0.846	0.537	0.952
	(neu, mas)	0.85783	0.779	0.528	40.71	0.848	0.857	0.556	0.953
SD v2.1									
GCC									
	(neu, fem)	0.980	0.755	0.522	41.48	0.842	0.805	0.527	0.952
	(neu, mas)	0.982	0.782	0.552	37.96	0.856	0.820	0.566	0.959
COCO									
	(neu, fem)	0.984	0.763	0.569	37.796	0.8670	0.858	0.556	0.955
	(neu, mas)	0.985	0.780	0.586	35.632	0.8747	0.853	0.575	0.957
TextCaps									
	(neu, fem)	0.9846	0.713	0.456	46.600	0.838	0.752	0.492	0.948
	(neu, mas)	0.9854	0.747	0.483	43.362	0.851	0.773	0.524	0.953
Flickr30k									
	(neu, fem)	0.982	0.772	0.499	42.722	0.853	0.823	0.544	0.9572
	(neu, mas)	0.983	0784	0.511	40.988	0.857	0.813	0.555	0.9570
Profession									
	(neu, fem)	0.85784	0.759	0.497	44.173	0.835	0.856	0.521	0.945
	(neu, mas)	0.85783	0.778	0.517	41.796	0.848	0.870	0.548	0.947

**Table 6:** Co-occurrence similarity on Stable Diffusion models.

Pairs		GCC	COCO	TextCaps	Flickr30k	Profession
SD v1.4						
	$s_{O}\left(P_{n},P_{f}\right)$	0.379	0.486	0.413	0.424	0.350
	$s_{O}\left(P_{n},P_{m}\right)$	0.414	0.516	0.444	0.457	0.374
SD v2.0						
	$s_{O}\left(P_{n},P_{f}\right)$	0.382	0.512	0.420	0.445	0.362
	$s_{O}\left(P_{n},P_{m}\right)$	0.425	0.531	0.448	0.476	0.376
SD v2.1						
	$s_{O}\left(P_{n},P_{f}\right)$	0.380	0.499	0.388	0.426	0.349
	$s_{O}\left(P_{n},P_{m}\right)$	0.419	0.522	0.419	0.451	0.382

**Table 7:** Chi-square test on dependency groups in each dataset on SD v1.4.

SD v1.4		Explicitly Guided	Implicitly Guided	Explicitly Independent	Implicitly Independent	Hidden
GCC						
	(neu, fem)	0.761	** $<{\mathrm{10}}^{-5}$ **	0.293	** $<{\mathrm{10}}^{-5}$ **	1
	(neu, mas)	0.607	** $<{\mathrm{10}}^{-5}$ **	0.805	** $<{\mathrm{10}}^{-5}$ **	1
	(fem, mas)	0.865	** $<{\mathrm{10}}^{-5}$ **	0.605	** $<{\mathrm{10}}^{-5}$ **	1
	Triplet	0.863	** $<{\mathrm{10}}^{-5}$ **	0.605	** $<{\mathrm{10}}^{-5}$ **	1
COCO						
	(neu, fem)	** $<{\mathrm{10}}^{-5}$ **	** $<{\mathrm{10}}^{-5}$ **	** $<{\mathrm{10}}^{-5}$ **	** $<{\mathrm{10}}^{-5}$ **	1
	(neu, mas)	** $<{\mathrm{10}}^{-5}$ **	** $<{\mathrm{10}}^{-5}$ **	** $<{\mathrm{10}}^{-5}$ **	** $<{\mathrm{10}}^{-5}$ **	1
	(fem, mas)	** $4\times {\mathrm{10}}^{-5}$ **	** $<{\mathrm{10}}^{-5}$ **	** $2\times {\mathrm{10}}^{-4}$ **	** $<{\mathrm{10}}^{-4}$ **	1
	Triplet	** $<{\mathrm{10}}^{-5}$ **	** $<{\mathrm{10}}^{-5}$ **	** $<{\mathrm{10}}^{-5}$ **	** $<{\mathrm{10}}^{-5}$ **	1
TextCaps						
	(neu, fem)	0.992	** $<{\mathrm{10}}^{-5}$ **	0.435	** $<{\mathrm{10}}^{-5}$ **	1
	(neu, mas)	0.990	** $<{\mathrm{10}}^{-5}$ **	0.905	** $<{\mathrm{10}}^{-5}$ **	1
	(fem, mas)	0.969	** $<{\mathrm{10}}^{-5}$ **	0.802	** $<{\mathrm{10}}^{-5}$ **	1
	Triplet	0.999	** $<{\mathrm{10}}^{-5}$ **	0.778	** $<{\mathrm{10}}^{-5}$ **	1
Flickr30k						
	(neu, fem)	0.654	** $<{\mathrm{10}}^{-5}$ **	0.297	** $<{\mathrm{10}}^{-5}$ **	1
	(neu, mas)	0.858	** $<{\mathrm{10}}^{-5}$ **	0.330	** $<{\mathrm{10}}^{-5}$ **	1
	(fem, mas)	0.858	** $<{\mathrm{10}}^{-5}$ **	0.650	** $<{\mathrm{10}}^{-5}$ **	1
	Triplet	0.812	** $<{\mathrm{10}}^{-5}$ **	0.277	** $<{\mathrm{10}}^{-5}$ **	1
Profession						
	(neu, fem)	0.598	** $<{\mathrm{10}}^{-5}$ **	0.288	** $<{\mathrm{10}}^{-5}$ **	1
	(neu, mas)	0.431	** $<{\mathrm{10}}^{-5}$ **	0.755	** $<{\mathrm{10}}^{-5}$ **	1
	(fem, mas)	0.380	** $<{\mathrm{10}}^{-5}$ **	0.497	** $<{\mathrm{10}}^{-5}$ **	1
	Triplet	0.428	** $<{\mathrm{10}}^{-5}$ **	0.299	** $<{\mathrm{10}}^{-5}$ **	1

**Table 8:** Chi-square test on dependency groups in each dataset on SD v2.0.

SD v2.0		Explicitly Guided	Implicitly Guided	Explicitly Independent	Implicitly Independent	Hidden
GCC						
	(neu, fem)	0.196	** $<{\mathrm{10}}^{-5}$ **	0.191	** $<{\mathrm{10}}^{-5}$ **	1
	(neu, mas)	0.878	** $<{\mathrm{10}}^{-5}$ **	0.690	**2 × 10^−3^**	1
	(fem, mas)	0.774	** $<{\mathrm{10}}^{-5}$ **	0.940	** $<{\mathrm{10}}^{-5}$ **	1
	Triplet	0.751	** $<{\mathrm{10}}^{-5}$ **	0.653	** $<{\mathrm{10}}^{-5}$ **	1
COCO						
	(neu, fem)	** $<{\mathrm{10}}^{-5}$ **	** $<{\mathrm{10}}^{-5}$ **	** $<{\mathrm{10}}^{-5}$ **	** $<{\mathrm{10}}^{-5}$ **	1
	(neu, mas)	**1.01 × 10 ^−5^**	** $<{\mathrm{10}}^{-5}$ **	**3.05 × 10 ^−5^**	** $<{\mathrm{10}}^{-5}$ **	1
	(fem, mas)	** $<{\mathrm{10}}^{-5}$ **	** $<{\mathrm{10}}^{-5}$ **	0.234	** $<{\mathrm{10}}^{-5}$ **	1
	Triplet	** $<{\mathrm{10}}^{-5}$ **	** $<{\mathrm{10}}^{-5}$ **	** $<{\mathrm{10}}^{-5}$ **	** $<{\mathrm{10}}^{-5}$ **	1
TextCaps						
	(neu, fem)	0.966	** $<{\mathrm{10}}^{-5}$ **	0.567	** $<{\mathrm{10}}^{-5}$ **	1
	(neu, mas)	0.992	** $<{\mathrm{10}}^{-5}$ **	0.897	**10^−4^**	1
	(fem, mas)	0.990	** $<{\mathrm{10}}^{-5}$ **	0.551	** $<{\mathrm{10}}^{-5}$ **	1
	Triplet	0.998	** $<{\mathrm{10}}^{-5}$ **	0.796	** $<{\mathrm{10}}^{-5}$ **	1
Flickr30k						
	(neu, fem)	0.638	** $<{\mathrm{10}}^{-5}$ **	0.174	** $<{\mathrm{10}}^{-5}$ **	1
	(neu, mas)	0.889	** $<{\mathrm{10}}^{-5}$ **	0.489	** $<{\mathrm{10}}^{-5}$ **	1
	(fem, mas)	0.541	** $<{\mathrm{10}}^{-5}$ **	0.897	** $<{\mathrm{10}}^{-5}$ **	1
	Triplet	0.704	** $<{\mathrm{10}}^{-5}$ **	0.391	** $<{\mathrm{10}}^{-5}$ **	1
Profession						
	(neu, fem)	0.232	** $<{\mathrm{10}}^{-5}$ **	0.857	** $<{\mathrm{10}}^{-5}$ **	1
	(neu, mas)	0.159	** $<{\mathrm{10}}^{-5}$ **	0.828	** $<{\mathrm{10}}^{-5}$ **	1
	(fem, mas)	0.643	** $<{\mathrm{10}}^{-5}$ **	0.684	** $<{\mathrm{10}}^{-5}$ **	1
	Triplet	0.235	** $<{\mathrm{10}}^{-5}$ **	0.929	** $<{\mathrm{10}}^{-5}$ **	1

**Table 9:** Chi-square test on dependency groups in each dataset on SD v2.1.

SD v2.1		Explicitly Guided	Implicitly Guided	Explicitly Independent	Implicitly Independent	Hidden
GCC						
	(neu, fem)	0.185	** $<{\mathrm{10}}^{-5}$ **	0.933	** $<{\mathrm{10}}^{-5}$ **	1
	(neu, mas)	0.573	** $<{\mathrm{10}}^{-5}$ **	0.826	**3 × 10^−4^**	1
	(fem, mas)	0.942	** $<{\mathrm{10}}^{-5}$ **	0.714	** $<{\mathrm{10}}^{-5}$ **	1
	Triplet	0.573	** $<{\mathrm{10}}^{-5}$ **	0.918	** $<{\mathrm{10}}^{-5}$ **	1
COCO						
	(neu, fem)	** $<{\mathrm{10}}^{-5}$ **	** $<{\mathrm{10}}^{-5}$ **	** $<{\mathrm{10}}^{-5}$ **	** $<{\mathrm{10}}^{-5}$ **	1
	(neu, mas)	** $<{\mathrm{10}}^{-5}$ **	** $<{\mathrm{10}}^{-5}$ **	** $<{\mathrm{10}}^{-5}$ **	** $<{\mathrm{10}}^{-5}$ **	1
	(fem, mas)	** $<{\mathrm{10}}^{-5}$ **	** $<{\mathrm{10}}^{-5}$ **	0.056	** $<{\mathrm{10}}^{-5}$ **	1
	Triplet	** $<{\mathrm{10}}^{-5}$ **	** $<{\mathrm{10}}^{-5}$ **	** $<{\mathrm{10}}^{-5}$ **	** $<{\mathrm{10}}^{-5}$ **	1
TextCaps						
	(neu, fem)	0.839	** $<{\mathrm{10}}^{-5}$ **	0.234	** $<{\mathrm{10}}^{-5}$ **	1
	(neu, mas)	0.994	** $<{\mathrm{10}}^{-5}$ **	0.467	** $<{\mathrm{10}}^{-5}$ **	1
	(fem, mas)	0.972	** $<{\mathrm{10}}^{-5}$ **	0.941	** $<{\mathrm{10}}^{-5}$ **	1
	Triplet	0.993	** $<{\mathrm{10}}^{-5}$ **	0.686	** $<{\mathrm{10}}^{-5}$ **	1
Flickr30k						
	(neu, fem)	0.186	** $<{\mathrm{10}}^{-5}$ **	0.361	** $<{\mathrm{10}}^{-5}$ **	1
	(neu, mas)	0.113	** $<{\mathrm{10}}^{-5}$ **	0.356	** $<{\mathrm{10}}^{-5}$ **	1
	(fem, mas)	0.539	** $<{\mathrm{10}}^{-5}$ **	0.926	** $<{\mathrm{10}}^{-5}$ **	1
	Triplet	0.109	** $<{\mathrm{10}}^{-5}$ **	0.504	** $<{\mathrm{10}}^{-5}$ **	1
Profession						
	(neu, fem)	0.428	** $<{\mathrm{10}}^{-5}$ **	0.677	** $<{\mathrm{10}}^{-5}$ **	1
	(neu, mas)	0.470	** $<{\mathrm{10}}^{-5}$ **	0.603	** $<{\mathrm{10}}^{-5}$ **	1
	(fem, mas)	0.263	** $<{\mathrm{10}}^{-5}$ **	0.677	** $<{\mathrm{10}}^{-5}$ **	1
	Triplet	0.338	** $<{\mathrm{10}}^{-5}$ **	0.703	** $<{\mathrm{10}}^{-5}$ **	1

**Table 10:** Representational disparities between the neutral, feminine, and masculine in the three spaces from intra-prompts (SD v2.0).

Pairs	Prompt	Denoising	Image					
	t	$z_{0}$	*SSIM* ↑	*Diff. Pix.* ↓	*ResNet* ↑	*CLIP* ↑	*DINO* ↑	*Split-Product* ↑
(neu, fem)	0.981	0.789	0.547	37.54	0.867	0.844	0.557	0.947
(neu, mas)	0.982	0.829	0.587	33.81	0.892	0.864	0.625	0.959

**Table 11:** The proportion of images containing the dependency groups to all the images for each dataset on SD v2.0.

Dataset	Explicitly Guided	Implicitly Guided	Explicitly Independent	Implicitly Independent	Hidden
GCC	64.48	90.70	7.81	59.11	96.14
COCO	83.67	93.54	10.47	57.53	92.61
TextCaps	61.97	86.60	8.78	61.90	99.10
Flickr30k	83.07	94.91	9.56	58.89	92.48
Profession	15.03	98.07	3.48	63.22	100.00

**Table 12:** Amount of individual objects in each dependency group and nouns in prompts on SD v2.0 for each dataset.

Dataset	Explicitly Guided	Implicitly Guided	Explicitly Independent	Implicitly Independent	Hidden	Nouns
GCC	155	1059	85	625	536	544
COCO	827	2418	391	1529	3274	3305
TextCaps	371	1347	147	741	3608	3638
Flickr30k	659	2017	330	1255	2718	2741
Profession	162	1331	76	650	1041	1043

**Table 13:** Intersection ratio of individual objects among dependency groups and nouns in the prompts on SD v2.0.

		Explicitly Guided	Implicitly Guided	Explicitly Independent	Implicitly Independent	Hidden	Nouns
GCC							
	Over Explicitly guided	100.00	76.77	48.39	66.45	94.84	100.00
	Over Implicitly guided	11.24	100.00	0.63	48.35	14.16	14.83
	Over Explicitly independent	88.24	78.82	100.00	75.29	92.94	100.00
	Over Implicitly independent	16.48	81.92	10.24	100.00	22.08	23.04
	Over Hidden	27.43	27.99	14.74	25.75	100.00	100.00
	Over Nouns	28.49	28.86	15.62	26.47	98.53	100.00
COCO							
	Over Explicitly guided	100.00	93.95	44.98	78.72	96.37	100.00
	Over Implicitly guided	32.13	100.00	15.67	58.35	45.16	46.36
	Over Explicitly independent	95.14	96.93	100.00	93.61	99.23	100.00
	Over Implicitly independent	42.58	92.28	23.94	100.00	52.71	54.35
	Over Hidden	24.34	33.35	11.85	24.62	100.00	100.00
	Over Nouns	25.02	33.92	11.83	25.14	99.06	100.00
TextCaps							
	Over Explicitly guided	100.00	86.79	32.88	60.92	91.91	100.00
	Over Implicitly guided	23.90	100.00	9.58	47.29	37.27	39.20
	Over Explicitly independent	82.99	87.76	100.00	76.87	95.24	100.00
	Over Implicitly independent	30.50	85.96	15.25	100.00	44.13	46.69
	Over Hidden	9.50	13.90	3.88	9.06	100.00	100.00
	Over Nouns	10.20	14.51	4.04	9.51	99.18	100.00
Flickr30k							
	Over Explicitly guided	100.00	92.56	44.76	73.90	96.81	100.00
	Over Implicitly guided	30.24	100.00	15.62	55.97	43.88	44.72
	Over Explicitly independent	89.39	95.45	100.00	86.97	97.27	100.00
	Over Implicitly independent	38.80	89.96	22.87	100.00	51.16	52.11
	Over Hidden	23.47	32.56	11.81	23.62	100.00	100.00
	Over Nouns	24.04	32.91	12.04	23.86	99.16	100.00
Profession							
	Over Explicitly guided	100.00	81.48	38.89	60.49	98.77	100.00
	Over Implicitly guided	9.92	100.00	4.73	42.15	14.12	14.27
	Over Explicitly independent	82.89	82.89	100.00	75.00	98.68	100.00
	Over Implicitly independent	15.08	86.31	8.77	100.00	20.31	20.62
	Over Hidden	15.37	18.06	7.20	12.68	100.00	100.00
	Over Nouns	15.53	18.22	7.29	12.85	99.81	100.00

## Data Availability

All images presented in the paper are generated by Stable Diffusion.
